# Capturing Interactive Work for Nurses—First Validation of the German IWDS-N as a Multidimensional Measure

**DOI:** 10.3390/ijerph18157786

**Published:** 2021-07-22

**Authors:** Jan Digutsch, Maria Velana, Gerhard Rinkenauer, Sabrina Sobieraj

**Affiliations:** 1 Department of Psychology and Neurosciences, Leibniz Research Centre for Working Environment and Human Factors at the Technical University Dortmund, 44139 Dortmund, Germany; 2 Department of Ergonomics, Leibniz Research Centre for Working Environment and Human Factors at the Technical University Dortmund, 44139 Dortmund, Germany; velana@ifado.de (M.V.); rinkenauer@ifado.de (G.R.); sobieraj@ifado.de (S.S.)

**Keywords:** interactive work, emotional labor, job demands, well-being, coping strategies

## Abstract

The theoretical framework of interactive work provides a multi-dimensional perspective on the interpersonal demands of nurses in nurse–patient interactions. It is defined by four dimensions: emotional labor directed to the self and others, cooperative work, and subjective acting. While the framework stems from qualitative research, the aim of the current study is to translate it into a quantitative scale to enable measurement of the high interpersonal demands that so often remain implicit. For this reason, we conducted an online survey study (*N* = 157; 130 women, 25 men, 2 divers) among professional nurses in Germany (spring 2021) to test the derived items and subscales concerning interactive work, which resulted in a 4-factor model that was verified with confirmatory factor analysis (CFA). The survey further captured additional information on established constructs concerning job-related well-being (e.g., burn out, meaningfulness), job characteristics (e.g., work interruptions, time pressure) and individual resources (coping strategies) that are supposed to correlate with interactive work demand scales for nurses (IWDS-N), to determine the quantitative nature of their relations. The results show that the subscales of the IWDS-N have adverse effects on indicators of work-related well-being. Moreover, negative job characteristics, such as time pressure, are positively correlated with subscales of the IWDS-N and are therefore problem-focused coping strategies as an individual resource. The results emphasize that a multidimensional consideration of self-regulatory processes is useful to capture the subtle and complex nature of the interactive work demands of nurses. The current study is the first that developed a quantitative, multi-dimensional measure for interactive work demands, which can help make implicit demands in service work explicit.

## 1. Introduction

Nurses play an integral role in each health care system and strive to create healing environments where they can use their skills and provide their service recipients with the best care [1]. In order to bring caring to health care systems, nurses are required to engage in emotional tasks that not only imply the emotional regulation of their deep feelings, but also highlight the need to build relationships with their care recipients based on mutual trust [2]. At the same time, they should comply with the rules for providing services defined by the organization [3]. At the heart of the nursing profession is the interaction with others [4], who have their own needs, interests, and expectancies towards the services provided by their nurses [5]. The integrated model of interactive work [6,7,8,9] claims that a service can only be achieved by the successful interaction between the service provider and the service recipient in that the interaction is not proceeded unilaterally by the service provider; the recipients are also actively involved in the process, making both parties interrelated. Therefore, the service recipient is not regarded as a mere “object” of the work, i.e., they are not a purely passive consumer. On the contrary, the recipient is included as a subject and co-producer in work activities [10].

According to Böhle et al. [7], interactive work is characterized by four pivotal demands: inner emotional labor, outer emotional labor, cooperative work, and subjective acting. Inner emotional labor refers to the conflict of the service provider between the emotions that are actually felt and the emotions that need to be displayed. Outer emotional labor relates to emotion regulation that is directed towards the service recipient. Cooperative work encompasses the establishment of a cooperative relationship with the service recipient and subjective acting refers to an intuitive acting in vague or uncertain situations. Therefore, interactive work demands are an inherent part of a nurse’s daily work life. Numerous qualitative studies relying on this integrated model identified that interactive work demands are linked with emotional and physical consequences, as well as work intensity (e.g., [4,7,11]). In addition, extensive quantitative research highlights the adverse effects of inner emotional labor on the indicators of work-related well-being, such as burnout or work engagement (e.g., [12,13,14]).

In our study, we seek to make two main contributions to the literature on interactive work. First, we aim to develop a quantitative measurement for the integrated model of interactive work by Böhle et al. [7]. So far, only qualitative measures have been used. Secondly, the way caregivers interact with each other and with their environment can be influenced by factors related to the individual and nature’s work. Therefore, we aim to gain insights into how interactive work relates to indicators of work-related well-being (e.g., burnout, fatigue, work engagement), job characteristics (e.g., job control), and different coping and management strategies within the category of individual resources.

### 1.1. Interactive Work Demands

The integrated model of interactive work has developed over many years (for the most recent review see [8]). The concept provides a multifaceted perspective on the service industry and how services can successfully be obtained through the interaction of services provided and the service recipients. Labor in the service industry is defined as interactive work, which is characterized by four pivotal, intertwined demands from the service provider’s side: inner emotional labor, outer emotional labor, cooperative work, and subjective acting. The wording of the first two dimensions is very nuanced in the language of origin, which poses the risk that dimensions in English will not be understood as distinct. For this reason, we decided to differentiate both dimensions with the addition of “inner” and “outer” to make the target of emotion management and regulation clear. We will further elaborate on the dimensions in the following sections.

Inner emotional labor is usually named emotional labor and refers to the management and regulation of one’s affects and emotions. When individuals perceive discrepancies between actual, authentic feelings on the one hand and expected feelings and emotional rules of the organization on the other hand, they experience emotional dissonance [15]. One example of this are flight attendants who have to smile to create an emotionally pleasant atmosphere. In comparison to the service sector in general, nursing additionally demands the management of supposed inappropriate emotions such as disgust, pity or grief [7]. Outer emotional work also appears to play an essential role in maintaining the relationship between nurse and patient. The nurse can convey to the patient a sense of worth or of being used, in order to balance the relationship. This is, in part, elemental to maintaining the relationship and is crucial for some people to be able to care for them at all. In addition, outer emotional work can function as a basis of cooperative work, in that empathy provides a means for negotiating interests [11].

Cooperative work focuses on the establishment of a cooperative relationship between service providers and service recipients to obtain the service. Both parties have to agree on the service and the process of obtaining it; agreement can be reached explicitly in talking about it, or implicitly, when the circumstances are highly normative (e.g., nobody expects psychotherapy at a fast-food restaurant). Disagreement on the service and the process can prevent successful service. Nevertheless, discrepancies in service expectations of service providers and service recipients cannot be fully ruled out. Service recipients are often not aware of this nor how they should and can contribute to the success of the service [8,16]. The more the service recipient is involved in service delivery, the greater the need for cooperation. In the context of nursing, nurses and patients must work together to achieve the service goal, such as daily body care, and the better the cooperation the better the achievement.

Subjective acting refers to the ability of service providers to intuitively react to uncertain situations. Subjective acting comes into its own in particular when it is necessary to act quickly in unplanned, unpredictable situations or to deal with imponderables. This seems to be particularly relevant in personal services, since working with and on people is fundamentally associated with imponderables and, for example, behavior and reactions cannot fully be planned in advance. This urges service providers to apply an explorative, dialogic-interactive approach, to trust their senses (e.g., odd smells or unusual sounds) and their experiential knowledge [7,8,17]. The demands of subjective acting easily translates into the nursing context when nurses have to react to situations, such as noticing odd smells during wound care.

### 1.2. Work-Related Well-Being as a Potential Consequence of Interactive Work Demands

While each dimension contributes to interactive work, the demands can be assumed as challenging and exhausting if required extensively. Therefore, we assume that the demands of interactive work are associated with work-related well-being outcomes, such as burn-out or meaningfulness. In addition, we anticipate that certain job characteristics as well as individual resources are intertwined with interactive work demands. Well-being is operationalized across the literature in various ways and in the current paper we refer to work-related outcomes that contribute to employee’s well-being. On the one hand, we use fatigue and burn-out (emotional exhaustion and depersonalization) as negative representations of well-being to cover emotional, mental, physical, and behavioral indicators of well-being. On the other hand, work engagement and meaningfulness represent the positive spectrum of work-related well-being.

#### 1.2.1. Negative Indicators of Work-Related Well-Being

Interactive work is a form of work that requires high levels of goal-directed, flexible, and volitionally controlled behavior [18]. The control of emotions, thoughts, and behavior required for this is referred to as self-control [19]. Accordingly, flexible, goal-directed control and adaptation, as well as control of behaviorally effective processes [20,21], is necessary to continually realign one’s own behavior with patient needs and organizational requirements. However, findings from psychological research indicate that exercising self-control comes at a cost [20] and can translate into both short-term (e.g., ego depletion, need for recovery; [22]) and long-term states of exhaustion (e.g., burnout, [23]; for a review, see [24]). These findings are mainly theoretically and are underpinned with the strength model of self-control [20]. This model is based on the central assumption that different forms of self-control claim the same limited regulatory resource (willpower). When self-control is exerted, this resource is claimed, causing it to be temporarily depleted and thereby causing performance losses in subsequent self-control. Research has provided compelling evidence that the exercise of inner emotional labor has adverse impacts on emotional, mental, physical, and behavioral indicators of work-related well-being (e.g., [12,13,25,26,27]).

We assume that the other three dimensions rely on similar self-control mechanisms. We therefore expect all interactive work subscales to be positively related to emotional exhaustion, depersonalization, and fatigue (mental and physical). All dimensions require a high degree of goal-directed, flexible, and volitionally controlled behavior and thus access, at least in part, the limited regulatory resource of willpower.

#### 1.2.2. Positive Indicators of Work-Related Well-Being

In contrast to negative representations of work-related well-being, Schaufeli and Bakker [28] define work engagement as a positive, work-related state in the individual characterized by vigor, dedication, and absorption. Vigor is characterized by a high level of energy and mental resilience during work and a willingness to exert oneself at work despite difficulties. Dedication refers to feeling important, enthusiastic, inspired, and challenged about one’s work. Absorption is characterized by full concentration as well as the feeling of being tied down by the work. We expect a negative relationship of work engagement with inner and outer emotional work. Given the unclear or nonexistent literature for cooperative work and subjective acting, we would predict no relationship between work engagement and cooperative work and work engagement and subjective acting as an initial hypothesis.

Similarly, meaningfulness is an integral part of work life, since it encourages employees to appraise their job as meaningful and concentrate on their tasks [29]. Work meaningfulness includes three primary facets: positive meaning that is one’s personal sense that what they are doing is charged with significance, meaning-making through work that helps individuals with the ability to perceive the world around them and cultivate meaningfulness through experiences at work, and greater good motivations that imply that work is appraised as more meaningful if it has a greater impact on another’s life. The literature suggests that those employees who consider their tasks meaningful are more likely to show high levels of work engagement and responsibility, even during times of crisis [30,31]. For example, nurses ascribing a strong meaning to their job might exhibit a higher degree of emotional labor during the COVID-19 pandemic so as to comply with the desired behaviors embedded in organizational culture. In contrast, employees who ascribe a low meaning to their work are more prone to be distracted by the difficulties emerging from a stressful event [31] and may need a longer recovery period afterwards [32].

Table 1 summarizes the hypothesized relationships between interactive work demands and work-related well-being.

### 1.3. Job Characteristics as Potential Predictors of Interactive Work Demands

#### 1.3.1. Work Interruptions

It is well-known that surrounding work conditions can affect work-related well-being of employees and consequently, their health status [33]. Being interrupted by others is a common phenomenon in modern workplaces. Although work interruptions, in some cases, can transmit important information or stimulate daily work routines [34], they are broadly considered to have a negative impact on employees. Traditionally, work interruptions are associated with physical complaints, emotional exhaustion, and distress among employees [35]. Apart from the high physiological and psychological workload, interruptions can lead to low-quality services. In some work settings (e.g., in aviation), interruptions are linked with error-prone decisions which can sometimes cause serious and fatal accidents [36]. In the health sector, Chisholm et al. [36] also revealed that physicians working in emergency departments faced roughly ten interruptions per hour, possibly affecting the quality of health care provision. Taking into consideration the stressful nature of clinical environments, one might argue that nurses’ uncontrolled workload, which can be interrupted any time by care-related critical activities, urges them to continuously shift their attention on different tasks, disrupting, however, their thought process and rendering them susceptible to medical errors. Equally, a constant feeling of not having enough time to execute all work tasks or being under pressure [37] can be a source of job-related stress in the nursing profession, which may also result in an increased perception of interactive work demands.

#### 1.3.2. Time Pressure

Gelsema et al. [38] examined how job demands, such as work and time pressure, could influence the health status and well-being of nurses. In fact, they indicated that psychological outcomes (i.e., psychological distress, physical complaints and emotional exhaustion) were strongly influenced by time and work pressure. On the other hand, it was suggested that less work and time pressure could improve job satisfaction and decrease emotional exhaustion. A later study investigating what could induce the most stress in nurses from European countries, as well as in the U.S., showed that time pressure was one of the most frequently mentioned factors of stress and anxiety among UK., Italian, and U.S. nurses [37]. In addition, a qualitative study conducted by Roth et al. [4] indicates that nurses with high interactive work demands experience more time pressure than nurses with low interactive work demands. Therefore, as pressure at work is implicated as a source affecting employees’ well-being and health, we expect that it would positively relate to the concept components of interactive work demands.

#### 1.3.3. Effort–Reward Imbalance

Equally, when employees perceive organizations’ decisions as unfair, it may generate strong negative emotions, such as anger and resentfulness [39]. Perceived injustice is thoroughly conceptualized by the model of an effort–reward imbalance (ERI; [40]). According to this theoretical approach, social reciprocity is a fundamental principle of any social exchange at work that implies mutual cooperative investments and expected rewards analogous to effort investment. Any attempt to violate the balance between effort and respective rewards can lead to poor health and sustained stress reactions [40] which, in turn, may cause undesired effects on the organization’s proper function [39]. For example, nurses who perceived a great imbalance between extrinsic efforts spent and extrinsic rewards obtained were more likely to report higher levels of emotional exhaustion and depersonalization [41]. In addition, higher effort–reward imbalance is significantly associated with depression and anxiety in nursery [42]. In line with this, it might be argued that the imbalance between invested effort and received reward might intensify negative emotions and the way they are expressed so as to be in accordance with organizational rules, leading to higher levels of inner and outer emotional labor. Similarly, employees who perceive an injustice at the workplace might frequently feel forced to utilize their own experience to overcome a job burden and deal with challenges. Given that effort–reward imbalance can hamper employees’ well-being and generally be a barrier to employees, we expected that it would positively relate to the demands of interactive work.

#### 1.3.4. Job Control

Although numerous job characteristics are considered to negatively influence job-related outcomes, there are concepts of occupational research that function as job resources and enable the achievement of job-related goals and well-being. Particularly, job control helps employees develop an active approach to their working environment and determine how tasks are executed in terms of time and method [43]. According to Jackson et al. [44], time control is an individual’s opportunity to define their own time schedule, and method control implies individuals’ authority to decide how tasks will be carried out. Previous research has proposed that job resources, such as job control, may ameliorate the effects derived from stress exposure on employees’ job well-being and health [45]. Job control may also change how potential stressors produce unpleasant emotions and in turn, how these emotions cause distress. In addition, Mackey and Perrewé [46] argue that future research should consider how job control affects this strain process. Based on these notions, one would assume that, when an intensive control of deep feelings and their external expression is required, it might be less likely for employees to exert control during job-time. This led us to assume that job control would negatively relate to the perceived demands of inner and outer emotional labor.

Table 1 summarizes the hypothesized relations between interactive work demands and job characteristics.

### 1.4. Individual Resources as Potential Predictors of Interactive Work Demands

Individuals who experience stressful events at work derived from job-related characteristics will possibly develop health problems and poor psychological well-being. In an attempt to overcome stress, individuals may activate different coping strategies depending on the situational characteristics, the individuals’ appraisals, and their resources available to handle the demanding situation [47]. According to Lazarus and Folkman [48], coping strategies for alleviating the impact of a stressor can be differentiated between problem-focused and emotion-focused coping. Problem-focused coping refers to responses directed to modify or change situational aspects. As suggested by Carver et al. [49], problem-focused coping embraces active coping, suppression of competing activities, planning, seeking of instrumental social support, and restraint coping. Emotion-focused coping aims at managing emotions or cognitions, without altering the stressor or other aspects of the situation. Emotion-focused strategies include acceptance, positive reinterpretation, emotional social support, and denial.

Teo et al. [50] showed that effective coping strategies helped nurses overcome the aftermath of organizational changes by reporting higher job satisfaction. However, they did not report what types of coping were particularly successful to deal with organizational tensions. Although coping can have a mediating effect on stressors and job-related variables, findings seem to be inconclusive. Following this, we expected that nurses’ problem-focused coping will positively relate to the perception of cooperative work and subjective acting demands. In addition, emotion-focused coping will positively relate to the perception of inner and outer emotional labor. However, we did not expect a significant relationship between problem-focused coping and emotional labor. One might claim that actively seeking solutions for job-related problems might effectively apply to situations that are associated with subjective acting, and consequently urge situational modifications. Similarly, one might expect that emotion-focused strategies would be more effective in situations that demand primarily emotion suppression. Therefore, we do not expect a significant relationship between emotion-focused coping and cooperative work or subjective acting.

Table 1 summarizes the hypothesized relations between interactive work demands and individual resources.

## 2. Materials and Methods

### 2.1. Research Design and Participants

In order to develop a quantitative scale for interactive work, we conducted a cross sectional survey study among nursing staff in Germany. Participants (*N* = 157) were recruited based on their professions as nurses including those working at hospitals, nursing homes, and other organizations that provide professional care work. In 2019, about 4.5 million people worked in nursing profession in Germany [51]. Participants were not compensated but had the chance to take part in a prize draw. The survey, which was promoted through multiple social media postings, was available from March 2021 to May 2021. In total, 157 participants (130 women, 25 men, 2 divers) finished the survey. They ranged in age from 22 to 63 years (M = 38.19; SD = 10.75) and experience as professionals from 2 to 47 years (M = 18.34; SD = 10.80). The majority have worked in hospitals (*n* = 122), followed by those in geriatric nursing (*n* = 19) and outpatient care (*n* = 9) and “something different” (*n* = 8). Eighty-four participants had full-time contracts, while 73 had part-time contracts. Moreover, they were indicated to work overtime 4.34 h/week (SD = 5.99) on average. Most participants work in North-Rhine Westfalia (45%), followed by Bavaria (14%) and Lower Saxony (9%). Except for Saxony-Anhalt, every German state had at least one representative.

### 2.2. Measures

In addition to the development of the IWDS-N, we captured different concepts that relate to job resources and coping styles/personal resources to provide more information on the concept of interactive work. We correlated the following concepts in order to address our hypotheses/research questions.

#### 2.2.1. Work-Related Well-Being

For every variable in the section below, the scale score was calculated as the average of the single-item scores.

Emotional exhaustion was assessed with eight items from the German translation [52] of the Maslach Burnout Inventory [53]. This burnout dimension refers to feelings of being emotionally overextended of emotional and physical resources resulting from the demands of one’s work. An exemplary item is “I feel emotionally drained by my work.”. Participants responded on a six-point Likert scale (1 = not at all; 6 = very strong).

Depersonalization was captured with six items from the German translation [52] of the Maslach Burnout Inventory [53]. This burnout dimension is characterized by a cynical attitude toward people with whom one has to interact at work. An exemplary item is “I became more callous toward people since I took this job.”. Participants responded on a six-point Likert scale (1 = not at all; 6 = very strong).

Fatigue was measured with 12 items of the German version [54] of the Three-Dimensional Work Fatigue Inventory (3D-WFI; [55]). Given the overlap between the emotional fatigue subscale and the burnout dimension of emotional exhaustion, we only used physical and mental fatigue from the inventory. Exemplary items are “How often did you feel physically exhausted within the last weeks?” (physical) and “How often did you feel mentally exhausted within the last weeks?” (mental). Participants responded on a six-point Likert scale (1 = never; 6 = always).

Work Engagement was assessed with nine items of Schaufeli et al. [28]. The scale consists of the subscales vitality, dedication, and absorption. An exemplary item is “When I am working, I forget everything else around me.”. Participants responded on a six-point Likert scale (1 = totally disagree; 7 = totally agree).

Meaningfulness was measured with the Work as Meaning Inventory (WAMI) by Steger et al. [31] One exemplary item is “I have a good sense of what makes my job meaningful.”. Agreements were provided on a five-point Likert scale (1 = absolutely untrue; 5 = absolutely true).

#### 2.2.2. Job Characteristics

Work Interruptions were captured with four items of Lin et al. [35] Participants indicated their agreement to statements such as “I am frequently interrupted by others.” on a four-point Likert scale (1 = strongly disagree; 5 = strongly agree).

Time Pressure was captured with three items of Prümper et al. [56] An exemplary item is “At work, I am often pressed for time.” Responses were provided on a five-point Likert scale (1 = not at all; 5 = fully).

Effort–reward-imbalance was measured with five items of Van Yperen et al. [39] Items include statements such as “You work yourself too hard considering your outcomes.“ Agreements were given on five-point Likert scales (1 = never; 5 = very often).

Job Control was assessed through the sub-scales timing (three items, e.g., “At work, I can set my own pace of work.”) and method control (three items, e.g., “At work, I can decide how to go about getting my job done.”) by Jackson et al. [44] Participants responded on a four-point Likert scale (1 = not at all; 4 = a great deal).

#### 2.2.3. Individual Resources

Coping Strategies were captured with the German version [57] of the COPE measures [49]. In line with Sonnentag [58], we measured four coping strategies representing problem-focused coping (active coping, planning, restraint coping, use of instrumental social support) and two strategies representing emotion-focused coping (denial, use of emotional social support). Responses were given on a four-point Likert scale (1 = not at all; 4 = fully).

### 2.3. Analytical Procedure

To develop an item pool, we first studied the literature and available measures for each dimension of the integrated model of interactive work by Böhle et al. [7] The subscale inner emotional labor shows great conceptual overlaps with emotional dissonance [59] and surface acting [60], two well-established concepts in psychological literature. Thus, we oriented ourselves on those concepts for the item pool development. To examine content validity of our interactive work measures, we asked two professionals with work experience in nursing occupations to evaluate our items. Both verified the content of our items, as well as their fit and clarity for nurses in general. Then, we reduced the number of items and examined construct validity with the help of exploratory factor analysis. Discriminant validity of all subscales were tested using confirmatory factor analysis. Then, convergent validity was tested by examining the relations between interactive work demands and indicators of work-related well-being.

## 3. Results

### 3.1. Development of the Interactive Work Scale for Nurses (IWS-N)

We selected and developed items taking the following criteria into account: (1) items needed to reflect the core definitions of each dimension rather than antecedent boundary conditions; (2) specific work setting terminology was avoided such that the scale would be applicable to all nursing contexts. All items were formulated as work demands that express whether a certain action or behavior is required by the job. Responses were made on a five-point Likert scale ranging from 1 (totally disagree) to 5 (totally agree).

To select the best-fitting items for each subscale, we examined item difficulty by evaluating item scores as indicated by mean, standard deviation, median, skewness, and kurtosis. Two strongly skewed items were excluded from further analyses. The means of all remaining items ranged from 3.14 to 4.46, all standard deviations exceeded 0.50, which is an indicator of adequate variability [61]. Mean and skewness values indicate a tendency for high scores for each dimension.

To examine the factor structure, an exploratory factor analysis (principal components analysis) was used with an oblique rotation (i.e., oblimin), as we assumed dependency among the four factors. This assumption is based on the significant conceptual overlap of all four factors as indicated by [7,8]. In line with our theoretical model, four factors were suggested by a parallel analysis, supporting the four-factor solution. We excluded items that did not meet the factor loading cut-off criterion of 0.30 [62]. To further optimize the scale length and the distinctiveness of each factor, we stepwise removed items with lower factor loadings. In the final set of 14 items, all items had a minimum pattern loading of |0.63| as no cross-loadings above |0.21| emerged (see Table 2). All factors explain 60.9% of the variance, with each factor explaining between 11.0% and 21.3% of the variance.

To test the discriminant validity of the subscales, we conducted a confirmatory factor analysis (CFA) with robust standard errors. We tested a four-factor model including the four dimensions as distinct factors. The fit indices for this model indicated an acceptable fit: χ^2^ (71) = 148.08, *p* < 0.001, comparative fit index (CFI) = 0.93, Tucker-Lewis index (TLI) = 0.91), root mean square error of approximation (RMSEA) = 0.09, standardized root mean square residual (SRMR) = 0.06. Afterwards, we integrated all dimensions into one common factor (χ^2^ (77) = 536.62, *p* < 0.001, CFI = 0.53, TLI = 0.44, RMSEA = 0.21, SRMR = 0.18). This model performed worse in comparison to the four-factor model (Δχ^2^ (6) = 222.48, *p* < 0.001). Moreover, all possible two-factor models (Δχ^2^ (5) ≥ 589.27, *p* < 0.001) and three-factor models (Δχ^2^ (3) ≥ 104.00, *p* < 0.001) performed worse than the four-factor model. All goodness-of-fit statistics are displayed in Table 3.

In summary, the results of the conducted CFAs provide further evidence of the scale’s internal structure. For further analyses, we used the unweighted means of all scale items as indicators for the respective scales. The complete set of items in both English and German can be found in the Appendix A.

### 3.2. Hypotheses Testing

Table 4 displays the descriptive statistics, internal consistencies (Cronbach’s alphas), and correlations between all study variables. All interactive work demand subscales correlated moderately with each other.

Work Engagement was negatively related to inner emotional labor. Emotional exhaustion was positively related to all subscales with the exception of cooperative work. Depersonalization was positively related with inner emotional labor but negatively related to cooperative work. Fatigue was positively related to all subscales with the exception of cooperative work. Meaningfulness was positively related to cooperative work and subjective acting but not to both inner and outer emotional labor. Taken together, analysis largely confirmed our hypotheses for work-related well-being with the exception of depersonalization.

Job Control was negatively related to both inner and outer emotional labor but not to cooperative work and subjective acting. Effort–reward imbalance, time pressure, and work interruptions were all positively related to all subscales with the exception of cooperative work. Again, analysis largely confirmed our hypotheses. However, the subscale cooperative work was not related to any job characteristic. Both problem- and emotion-focused interpersonal emotion management were positively related to all subscales with the exception of subjective acting. Whereas problem-focused coping was positively related to all subscales, emotion-focused coping was positively related to inner emotional labor and cooperative work but not outer emotional labor and subjective acting.

Taken together, the overall pattern of correlations supported our hypotheses. Exceptions have to be made for the hypothesized relations between the subscale cooperative work and emotional exhaustion, fatigue, effort–reward imbalance, time pressure, and work interruptions which have not been found in our data. For depersonalization, the opposite relation (negative instead of positive) has been found.

Additionally, we tested whether the four dimensions of interactive work differ regarding the sociodemographic variables age, gender, employment type (full- vs. part-time), tenure (in years), and type of care facility (hospital, outpatient or stationary care facilities, other). Results indicate that the higher the age, the lower inner emotional labor demands nurses experience (r = 0.02, *p* < 0.05). For outer emotional labor, women report more higher demands than man (t = 2.63, df = 46.47, *p* < 0.05). For all other variables, no significant differences were found.

## 4. Discussion

The aim of the current study was to develop a scale for interactive work to make implicit work demands of emotional labor, cooperative work and subjective acting measurable. Based on the literature of interactive work we derived items that captured the core aspects of interactive work in the context of nursing, resulting in the interactive work demand scale for nurses (IWDS-N). In addition, we explored associations of potential outcomes concerning work-related well-being and antecedents referring to job resources and individual resources. The results show that we were available to obtain an interactive work scale with four distinct dimensions that obtained good scale metrics. The subscale outer emotional labor, as well as inner emotional labor, obtained good reliability. Since both dimensions referring to emotion regulation and emotional dissonance are well examined as constructs in work-related research settings (e.g., [15,63,64]), it is not astonishing that the majority of our hypothesized expectations were met.

Referring to work-related well-being, we found that the perceptions of outer emotional labor are associated with negative work outcomes as hypothesized, in that increased demand perceptions of inner emotional labor are positively correlated with symptoms of burnout and fatigue, while work engagement is negatively correlated. This is in line with former findings by, e.g., [12,27]. Moreover, the results revealed the assumed relations with job characteristics respectively. So, the higher the imbalance of invested effort in the job, the higher the time pressure on the job, the more work interruptions occur and the lower the perceived control on how and when single tasks are conducted, the higher the perceptions of managing one’s own emotional states. In addition, we found associations between individual resources focusing on strategies to overcome obstacles or problems. We found evidence of the hypothesized positive correlations between the perception of inner emotional labor and emotion-directed coping strategies. In an exploratory manner we found that problem-based coping is also positively correlated with inner emotional labor. It seems that no matter which strategy is applied, independent of the question of whether the strategy is affective or behavioral in nature, it goes along with intensified perceptions of emotional regulation demands. Thus, it seems that coping increases awareness for problem-solving and emotional demands likewise.

Outer emotional work showed a similar pattern of results. However, we could not find the predicted negative associations with work engagement, nor the positive associations with depersonalization, which was rather astonishing. We assumed that the more participants depersonalize from their patients, the higher are the perceived demands to manage patients’ emotions, because patients would rather be perceived as objects that need to be managed in order to get the job done. However, this was not the case in the current sample; participants seem to dissociate oneself from this idea and perceive their patients as subjects and managing others’ emotions as part of their job, which could also act as an explanation for the missing negative links between work engagement and outer emotional labor. Nevertheless, we found the negative associations between detrimental job characteristics and the perceived demands of outer emotional labor. The strains that are experienced through time pressure, work interruptions, low job control and an effort–reward imbalance seem to translate into an intensified perception of the demand to manage patients’ emotions. This is also in line with former research (e.g., [14,37,41]).

As predicted, we found that strategies of interpersonal emotion management are positively correlated with the demand of managing others’ emotions. In contrast to our assumptions, which were exploratory in nature, we found a positive relation in terms of problem-focused coping and no correlation with emotion-focused coping with the need to manage the emotions of others. We are inconclusive about the missing link between outer emotional labor and emotional coping, which refer to the same emotional resource in the individual. However, we assume that the association of outer emotional labor with behavioral coping strategies could be explained by the active character of outer emotional labor, since it requires behavioral action, whether it is about telling a joke to enlighten patients or merely about smiling at patients.

Since both emotional subscales are well examined as constructs in work-related research settings, it is not astonishing that the majority of our hypothesized expectations were met. In contrast, the subscales cooperative work and subjective acting are dimensions that have not been operationalized as of now, which shaped the exploratory process of item construction and the assumptions concerning relations with other constructs. However, both scales obtained good scale metrics; the exploratory nature is, indeed, mirrored by our findings.

The sub-dimension of cooperative work refers to the collaboration, which is needed between a service provider and the service receiver to achieve the service. The analysis revealed mixed results concerning the relation of demand to actively engage in cooperative work and work-related well-being concepts. As proposed, we found positive associations between cooperative work and the perception of meaningfulness. Further research needs to untangle whether the demand of cooperative work can be the source of meaningfulness or vice versa, that those striving for meaningfulness chose jobs with high cooperative work demands.

Contrary to our assumptions, we found that the less depersonalization the more cooperative work demands are perceived. While we originally suggested that objectifying patients would lead to an intensified perception of cooperative work in terms of a forced strain that becomes more salient, the analysis showed the opposite relation. The more patients are seen as individuals who are subject to empathy with their own needs, the higher the urge to engage in cooperative work. Again, it seems that our participants highly protected their ideas of patients in need, which is part of their job. This also resonates with the null correlations found for emotional exhaustion and fatigue. Future studies should address this and examine which other concepts, such as personality traits (e.g., altruism) may mediate this relationship.

The job characteristics were not all correlated with the demands of cooperative work, indicating that the subscale of cooperative work is independent from job control, effort–reward imbalance, time pressure and work interruptions. Compared with these individual resources in the form of problem-focused and emotion-focused coping strategies are positively correlated with cooperative work demands. The salience for dealing with problems or difficulties seems to be intertwined with the demands of engaging in cooperative work. Maybe this could hint at the way cooperative work is initiated, that it can be achieved through emotional strategies or behavioral strategies. Future studies should further examine the underlying processes.

The demands of Subjective Acting as the fourth sub-dimension of interactive work refers to the perception of how much employees trust their senses, deal with uncertainty and refer to their implicit (professional) knowledge. The analysis reveals positive correlations with emotional exhaustion, fatigue and meaningfulness. This indicates that the perceived demands of subjective acting are not entirely perceived as something that goes along with higher fatigue or emotional exhaustion; rather, it is associated with meaningfulness, which represents a positive outcome of work-related well-being. According to findings on the other three dimensions, our assumption of a positive relation between depersonalization and subjective acting needs to be rejected, since we found no significant correlation at all, such as for outer emotional labor. In contrast, we found evidence for all predicted correlations concerning job characteristics, indicating detrimental relationships. In terms of individual resources, the analysis confirmed our assumptions towards coping strategies; that is, problem-based coping is related to subjective acting while emotion- based coping is not.

### 4.1. Theoretical and Practical Implications

Compared to other concepts and frameworks that focus on a single construct or a domain of constructs, the interactive work model incorporates four distinct subdimensions that refer to emotion regulation demands, concerning one’s owns emotions and others’ emotions, demands that aim to create a cooperative relationship and subjective acting that refers to trust in one’s own senses and knowledge. From a theoretical perspective we added information on how interactive work relates to concepts commonly used by scholars in organization psychology, sociology or communication, such as fatigue and meaningfulness with work-related well-being, job control boundaries within the job characteristic category and different coping strategies within the category of individual resources. The results revealed that relationships to these additional constructs pose differently depending on the particular subscale. Therefore, the complexity of relationships emphasizes the need to consider multiple dimensions to estimate the demanding or elevating nature of service work. However, it has to be acknowledged that the presented correlations are descriptive, since the method did not allow for further testing of causality. This is subject to further empirical testing; cross-validations with different samples in different countries are needed to prove the validity of the scale.

In developing the IWDS-N, we strived to make a concept measurable that was an exclusive qualitative concept beforehand so that this valuable, multi-dimensional concept could be easily applied to a broader range of branches and occupations in service-work. Since the demands of interactive work used to stay implicit rarely become appreciated and remunerated, the scale could help to make these demands explicit. Moreover, this could inspire a systematic categorization of jobs in service work that require outer and inner emotional labor, cooperative work and subjective acting.

### 4.2. Limitations and Suggestions for Future Research

Our study has some limitations. The study provided first evidence for a valid multidimensional measurement, this implies that we could not compare our data and measurements with former studies, since the framework of interactive work was an exclusive qualitative approach. The convergent and divergent validity; therefore, they should be tested in future studies. Concerning the study conduct we did not focus on extended pilot testing with the target group; however, we derived the items for the IWDS-N from the qualitative material on interactive works and especially from recurrent declarations by nurses. In addition, the items were reviewed and approved by professionals in nursing and medicine in advance; afterwards, the questionnaire was pretested numerous times by the authors to ensure effective survey operation. As of now, we could not provide test–retest reliability; future studies could help to address this limitation and could benefit from a larger sample size to increase the power of the findings.

Moreover, our reported relations between two study variables are zero-order correlations that do not imply causality. It remains to be further proven whether interactive work demands impair indicators of work-related well-being or vice versa. Even if the strong conceptual overlap between the subscale inner emotional labor and emotional dissonance constitutes a strong clue for causality (e.g., [15]), we suggest longitudinal studies to validate this assumption. The current study considers direct effects between interactive work demands and potential outcomes and predictors. Given the extensive research on moderators and mediators affecting the relationship between job demands and its outcomes (e.g., [22,65]), the reported relations should be interpreted with caution. We highly encourage scholars to examine the underlying mechanisms of interactive work and the conditions affecting its consequences. Prior research indicates substantial day-specific fluctuations of emotional dissonance, which has led researchers to conduct multi-level analyses (e.g., [12,13]). For this reason, future research should distinguish between day- and person-level variance of interactive work demands.

Another improvement could address the measures used, especially the measurement of coping styles: we recommend using a different measurement of emotional- and problem-based coping styles, which may obtain better reliability metrics and, hence, more statistically powerful results. In addition, we did not use a validated German adaptation for the scales work engagement, meaningfulness, work interruptions, effort–reward imbalance and job control. Although self-report questionnaires were absolutely sufficient for the purpose of the current study, future projects could use, for example, physiological and behavioral measures that provide more objective data. When considering empirical studies suitable for field studies in the nursing context (e.g., [66,67,68,69]), for example, heart rate (HR); heart rate variability (HRV); locomotor activity; and cortisol would be obvious indicators to identify the workload. In order to deal with the difficulty in measuring mental stress, HR and HRV are considered parameters of general activation. These parameters allow us to describe the vegetative balance of the organism and through the corresponding stress parameters, conclusions can be drawn about previous mental stress experiences. In addition, HRV can be used as an indicator of psychophysical states of the organism and as an indicator of the limitations of an adaptive capacity with respect to stress (for an overview with respect to HR and HRV, see Sammito et al., [70]). With respect to psychological stress, there are emerging methods that allow the identification of episodes of non-metabolic HRV reduction as an indicator of psychological stress in everyday life; for example, by taking into account locomotor activity [71]. In addition, the stress hormone cortisol (surveyed via saliva samples) could also provide important information about psychological stress and recovery processes [66].

### 4.3. Conclusions

The concept of interactive work is correlated with different job-related well-being constructs. However, correlation directions need to be differentiated for each subscale; not every relation is shaped in the same way. The same is true for job characteristics and individual resources and the four subscales of interactive work. Further research is needed to determine the exact nature of relationships and whether our tentative assumptions that job characteristics and individual resources are potential predictors of interactive work and work-related well-being as an outcome of interactive work are correct. Moreover, since we have conducted the study in Germany, the items and constructs should become subject to international examination to determine their validity. The framework of interactive work is unique in its combination of four sub-scales that go beyond emotional demands of service work. It gives the chance to make service work, with its complex inherent demands that otherwise stay disregarded and unpaid, quantifiable and valuable.

## Figures and Tables

**Table 1:** Hypothesized Relations Between Interactive Work Demands, Psychological Well-Being, Job Resources, and Individual Resources.

Variables	Emotional Labor		Cooperative Work	Subjective Acting
	Inner	Outer		
Work-Related Well-Being				
Emotional Exhaustion	+	+	+	+
2. Depersonalization	+	+	+	+
3. Fatigue	+	+	+	+
4. Work Engagement	-	−	0	0
5. Meaningfulness	0	0	+	+
Job characteristics				
6. Work Interruptions	+	+	+	+
7. Time Pressure	+	+	+	+
8. Effort-reward-imbalance	+	+	+	+
9. Job Control	−	−	0	0
Individual resources				
10. Problem-focused Coping	−	−	+	+
11. Emotion-focused Coping	+	+	0	0

Note. + = Hypothesized positive relation; − = hypothesized negative relation; 0 = no relation hypothesized.

**Table 2:** Factor Loadings and Alphas for Interactive Work Demands Measures.

Item	Emotional Labor		Cooperative Work	Subjective Acting
	Inner	Outer		
I have to display feelings that do not match with what I actually feel toward the patients.	0.88			
I have to show feelings in my interactions with patients that do not correspond with the feelings that I actually experience.	0.93			
I have to endure conflicts between my own feelings and the feelings I should show toward the patients.	0.80			
I have to express certain feelings that I don’t actually feel.	0.84			
I always have to establish a positive atmosphere when interacting with patients.		0.71		
I have to help patients cope with negative feelings (e.g., anxiety, sadness).		0.65		
I have to be good at comforting patients.		0.71		
I have to team up with the patients to achieve positive outcomes.			0.66	
I have to involve the patients in my work.			0.79	
I have to be an attachment figure for the patients.			0.76	
I have to maintain a trusting relationship with the patients.			0.63	
I have to pay close attention to the body language of the patients.				0.68
I have to read between the lines during interactions with patients.				0.82
I have to actively draw on my sensations during interaction with the patients.				0.86
Cronbach’s alpha	0.92	0.76	0.81	0.84

*Note.* Only factor loadings > 0.30 are shown.

**Table 3:** Goodness-of-Fit Statistics.

Model	χ^2^	df	CFI	TLI	RMSEA	SRMR
One-factor model	536.62	77	0.53	0.44	0.21	0.18
Best fitting two-factor model ^a^	384.15	76	0.72	0.66	0.17	0.12
Best fitting three-factor model ^b^	240.09	74	0.84	0.81	0.13	0.10
Four-factor model	148.08	71	0.93	0.91	0.09	0.06

Note: CFI = comparative fit index; TLI = Tucker-Lewis index; RMSEA = root mean square error of approximation; SRMR = standardized root mean square residual. ^a^ Emotional Labor (Inner and Outer) items loading on the first factor, Cooperative Work and Subjective Acting items loading on the second factor. ^b^ Emotional Labor (Inner and Outer) items loading on the first factor, Cooperative Work items loading on the second, and Subjective Acting items loading on the third factor.

**Table 4:** Means, Standard Deviations, Zero-Order Pearson-Correlations, and Alphas of all Study Variables.

Variable	M	SD	1	2	3	4	5	6	7	8	9	10	11	12	13	14	15
Interactive Work Demands																	
Emotional Labor (inner)	3.42	1.13	0.92														
2. Emotional Labor (outer)	4.21	0.68	0.43	0.76													
3. Cooperative work	4.39	0.72	0.20	0.21	0.81												
4. Subjective Acting	4.13	0.80	0.37	0.36	0.39	0.84											
Work-Related Well-Being																	
5. Emotional Exhaustion	3.73	1.20	0.37	0.28	0.04	0.19	0.91										
6. Depersonalization	2.66	1.17	0.26	0.06	−0.17	−0.02	0.53	0.85									
7. Fatigue	3.63	0.85	0.26	0.26	0.05	0.24	0.78	0.40	0.95								
8. Work Engagement	4.47	1.14	-0.23	−0.07	0.00	−0.05	−0.52	−0.48	−0.37	0.90							
9. Meaningfulness	3.73	0.73	-0.03	0.03	0.26	0.19	−0.27	−0.37	−0.11	0.50	0.83						
Job characteristics																	
10. Work Interruptions	3.96	0.87	0.31	0.17	0.08	0.25	0.49	0.25	0.38	−0.25	−0.09	0.89					
11. Time Pressure	4.09	0.91	0.37	0.40	0.15	0.29	0.58	0.26	0.47	−0.22	−0.09	0.52	0.82				
12. Effort–reward imbalance	3.67	0.97	0.31	0.33	−0.04	0.10	0.69	0.36	0.59	−0.40	−0.15	0.38	0.55	0.86			
13. Job Control	2.42	0.78	−0.26	−0.23	0.14	−0.08	−0.26	−0.30	−0.27	0.24	0.05	−0.20	−0.31	−0.26	0.89		
Individual resources																	
14. Problem-focused Coping	2.73	0.38	0.17	0.24	0.30	0.25	0.13	−0.15	0.16	0.04	0.21	0.17	0.18	0.06	0.00	0.48	
15. Emotion-focused Coping	2.40	0.57	0.18	0.09	0.16	0.09	0.16	−0.03	0.13	−0.08	0.11	0.03	0.07	0.17	−0.03	0.41	0.35

*Note.* Alphas are displayed on the diagonal. *N* = 157; all correlations r ≥ |0.16| are significant with *p* < 0.05.

## Appendix A

**Table A1:** English and German Items of the IWDS-N.

Emotional Labor (Inner)
1. I have to display feelings that do not match with what I actually feel toward the patients. *Ich muss nach außen hin Gefühle zeigen, die nicht mit dem übereinstimmen, was ich den zu Pflegenden gegenüber tatsächlich fühle.* 2. I have to show feelings in my interactions with patients that do not correspond with the feelings that I actually experience. *Ich muss im Umgang mit den zu Pflegenden Gefühle zeigen, die meinen eigentlich erlebten Gefühlen nicht entsprechen.* 3. I have to endure conflicts between my own feelings and the feelings I should show toward the patients. *Ich muss Konflikte zwischen meinen eigenen Gefühlen und den Gefühlen, die ich nach außen hin/den zu Pflegenden gegenüber zeigen sollte, aushalten.* 4. I have to express certain feelings that I don’t actually feel. *Ich muss bestimmte Gefühle zum Ausdruck bringen, die ich eigentlich nicht empfinde.*
Emotional Labor (outer)
5. I always have to establish a positive atmosphere when interacting with patients. *Ich muss im Umgang mit den zu Pflegenden stets eine positive Stimmung herstellen.* 6. I have to help patients cope with negative feelings (e.g., anxiety, sadness). *Ich muss den zu Pflegenden helfen, negative Gefühle (z.B. Ängste, Traurigkeit) zu bewältigen.* 7. I have to be good at comforting patients. *Ich muss den zu Pflegenden gut zusprechen können.*
Cooperative work
8. I have to team up with the patients to achieve positive outcomes. *Ich muss mit den zu Pflegenden zusammenarbeiten, um ein gutes Ergebnis zu erzielen.* 9. I have to involve the patients in my work. *Ich muss die zu Pflegenden in meine Arbeit mit einbinden.* 10. I have to be an attachment figure for the patients. *Ich muss eine Bezugsperson für die zu Pflegenden sein.* 11. I have to maintain a trusting relationship with the patients. *Ich muss eine vertrauensvolle Beziehung zu den zu Pflegenden pflegen.*
Subjective Acting
12. I have to pay close attention to the body language of the patients. *Ich muss sehr auf die Körpersprache der zu Pflegenden achten.* 13. I have to read between the lines during interactions with patients. *Ich muss während der Interaktion mit den zu Pflegenden zwischen den Zeilen lesen.* 14. have to actively draw on my sensations during interaction with the patients. *Ich muss während der Interaktion mit den zu Pflegenden aktiv auf meine Sinneseindrücke zurückgreifen*.

Note. Only the German items have been used in this study. The English translations are for documentation purposes only and may benefit of professional translation and validation of an English-speaking sample.
